# Roles of histone acetylation sites in cardiac hypertrophy and heart failure

**DOI:** 10.3389/fcvm.2023.1133611

**Published:** 2023-03-15

**Authors:** Masafumi Funamoto, Masaki Imanishi, Koichiro Tsuchiya, Yasumasa Ikeda

**Affiliations:** ^1^ Department of Pharmacology, Institute of Biomedical Sciences, Tokushima University Graduate School, Tokushima, Japan; ^2^ Department of Medical Pharmacology, Institute of Biomedical Sciences, Tokushima University Graduate School, Tokushima, Japan

**Keywords:** epigenetics, histone post-translational modification, cardiac hypertrophy, heart failure, acetylation

## Abstract

Heart failure results from various physiological and pathological stimuli that lead to cardiac hypertrophy. This pathological process is common in several cardiovascular diseases and ultimately leads to heart failure. The development of cardiac hypertrophy and heart failure involves reprogramming of gene expression, a process that is highly dependent on epigenetic regulation. Histone acetylation is dynamically regulated by cardiac stress. Histone acetyltransferases play an important role in epigenetic remodeling in cardiac hypertrophy and heart failure. The regulation of histone acetyltransferases serves as a bridge between signal transduction and downstream gene reprogramming. Investigating the changes in histone acetyltransferases and histone modification sites in cardiac hypertrophy and heart failure will provide new therapeutic strategies to treat these diseases. This review summarizes the association of histone acetylation sites and histone acetylases with cardiac hypertrophy and heart failure, with emphasis on histone acetylation sites.

## Introduction

Heart failure (HF), which occurs in the late stage of most cardiovascular diseases (CVDs), has a high mortality rate and worsens the patient life expectancy. The proportion of the population affected by HF is estimated to increase annually, placing a significant burden on public health in an aging society (1). Various CVDs can lead to HF *via* pathological cardiac hypertrophy. Cardiac hypertrophy is an important process of cardiac remodeling that is often caused by preload, such as hypertension, or afterload, such as myocardial infarction (2, 3). Cardiac hypertrophy is a compensatory mechanism that reduces oxygen consumption, normalizes the ventricular systolic pressure, and improves the ejection function. However, long-term stress, such as hypertension, eventually leads to HF and irreversible pathological cardiac remodeling. Cardiac hypertrophy also causes fibrosis and dysfunction, leading to HF (4, 5). Various biological regulatory processes are involved in cardiac hypertrophy and remodeling. Particularly, acetylation and deacetylation of histones *via* epigenetic modifications have attracted researchers' attention (6, 7). Histone acetylation and deacetylation are believed to play important roles in the regulation of gene expression, leading to cardiac hypertrophy and HF under stress. Histone acetylation is important for cardiac physiology and pathophysiology (8). However, the different sites of histone acetylation are not clearly understood. This review summarizes the potential mechanisms by which the differences in histone acetylation sites in the regulation of gene expression may be involved in the etiology of cardiac hypertrophy and HF. This review provides a better understanding of the regulatory mechanisms of histone acetylation in the pathogenesis of HF and suggests new therapeutic strategies for HF.

## Histone acetylation in epigenetics

Epigenetics is currently one of the fastest-growing areas in biological research. Epigenetics plays an important role in many cellular processes, including regulation of gene expression and transcription, cell growth and differentiation, and chromosome remodeling and inactivation (8, 9). Epigenetics refers to the regulation of gene expression *via* reversible chemical modifications of the DNA, histones, and even chromatin structures, without changing the DNA sequence. Epigenetic processes primarily include DNA methylation, non-coding RNAs, and histone modifications (10). The nucleosome is composed of 147 bp of DNA wrapped around an octamer containing four histone proteins: H2A, H2B, H3, and H4 (11). The linker histone protein H1 mediates interactions between adjacent nucleosomes, resulting in chromatin formation (12). Histones are characterized by their direct binding to DNA, and post-translational modifications (PTMs) of histones alter the chromatin structure and dynamically regulate the transcriptional state of genes (13). Chromatin is of two types: heterochromatin, which is closed and compressed, and euchromatin, which is relaxed and more suitable for transcription. In general, histone PTMs are controlled by “writers” that add modifier groups, “erasers” that remove modifier groups, and “readers” that specifically recognize the modification sites for downstream transcriptional activation or repression. All these PTMs are regulated by enzymes, and their enzymatic activity may be controlled by reactive cofactors, such as substrates and metabolic intermediates (14). Histone modifications include acetylation, methylation, butyrylation, propionylation, formylation, succinylation, malonylation, 2-hydroxyisobutyrylation, *β*-hydroxybutyrylation, glutarylation, benzoylation, and crotonylation (13, 15, 16). Among all histone modifications, acetylation and methylation are mostly shown to be involved in the regulation of CVDs, and other modifications, such as succinylation, have also been extensively studied in various diseases (15, 16). Histone acetylation is associated with the upregulation of gene transcription *via* various mechanisms, some of which are described below. First, PTMs of histone lysine residues alter the positive charge of the *ε*-amino group, which reduces nucleosome folding and affects DNA–histone or histone–histone interactions, thereby reducing the euchromatin–nucleosome interactions. However, interactions between enhancers and their corresponding promoters are strengthened (14, 17–19). Thus, PTMs of histones directly affect the structures of chromatin and nucleosomes. Second, PTMs of lysine residues on histones can serve as epigenetic landmarks directly or indirectly by the presence of transcription factors, and RNA polymerase II, which directly or indirectly mobilizes the acetylation of histone lysine residues, serves as an epigenetic landmark that is specifically recognized by transcription factors and chromatin remodeling factors. The acetylation level of lysine residues depends on the dynamic regulation of histone acetyltransferases (HATs) and histone deacetylases (HDACs) (14, 20). Acetylation sites of histones have mainly been studied for H3 and H4. Acetylation sites on histones H3 and H4 include H3K4, H3K9, H3K14, H3K18, H3K23, H3K27, H3K36, H3K56, H4K5, H4K8, H4K12, H4K16, and H4K20 (13, 21). In this review, we mainly focused on the acetylation of histones H3 and H4 and their involvement in cardiac hypertrophy and HF.

## Histone acetylation in cardiac hypertrophy and heart failure

The influence of epigenetics on disease incidence has drastically increased in the field of cancer, and in recent years, it has gradually expanded to almost all diseases, including HF (22, 23). Histone acetylation plays an important role in histone modification and affects heart diseases (22). Many histone acetylation sites have been suggested to be associated with heart diseases. Here, we discuss the relationship between histone acetylation sites and cardiac hypertrophy and HF.

Acetylation of H3K4 by p300 has been suggested to be important for the expression of the transcription factor, GATA-binding protein 4 (GATA4), in heart formation (24). Acetylation of H3K9 and H3K27 has also been suggested to be involved in the expression of GATA4, as GATA4 is involved in the development of HF and the fetal gene is reactivated during HF, suggesting that the acetylation of H3K4 may be involved in cardiac hypertrophy and HF (25, 26). Many reports have suggested that the acetylation of H3K9 by p300 causes cardiomyocyte hypertrophy (3, 4, 27–30). Furthermore, H3K9 acetylation is increased during cardiac hypertrophy and HF in TAC and Dahl rats (4, 26, 31). Acetylation of H3K9 is reduced by inhibition of the HAT activity of p300 (31, 32). Although some studies have suggested an association between H3K14 acetylation and microRNA (miR)-134–5p expression in cardiac fibroblasts, the specific role of H3K14 remains unknown due to the lack of comparisons with the normal state (33). Acetylation of H3K14 at the miR-30a-5p promoter in H9c2 cells is associated with c-Myc expression (34). However, whether H3K14 acetylation is directly involved in cardiac hypertrophy and HF remains unclear. Acetylation of H3K27 has been reported to be associated with cardiac disease, although not to the same extent as the acetylation of H3K9. Acetylation of H3K27 leads to cardiac hypertrophy and fibrosis of the heart together with bromodomain-containing 4 (35). In addition, H3K27 acetylation is associated with enhancers that are specifically activated in cardiac hypertrophy, and specific epigenetic signatures have been shown to regulate gene expression in hypertrophy by controlling promoter activity (36). Brd4 is a reader protein for H3K27 acetylation in CVDs (37, 38). Acetylation of H3K27 and H3K9 has also been shown to be a therapeutic target for CVDs (39). These acetylation sites are located in the tail domain of histones and implicated in cardiac hypertrophy and HF. Moreover, H3K122, a globular domain in the body of histones around which the DNA wraps, has also been reported to be involved in HF. In the Dahl rat model of hypertension-induced HF, acetylation of H3K9 increases during cardiac hypertrophy when the heart function is preserved, and acetylation of H3K122 in the globular domain increases during HF when the heart function is reduced. These histone acetylations are altered in the hypertrophic response gene promoter region and the recruitment of p300 remains unchanged during cardiac hypertrophy and HF. However, the binding of p300 to BRG1, a chromatin remodeling factor, increases during HF, and the recruitment of BRG1 to the promoter increases during cardiac hypertrophy. The mRNA levels of hypertrophic response genes are greatly increased in HF than in cardiac hypertrophy. This suggests that the acetylation of H3K122 in the histone body is involved in the pathogenesis of HF. Moreover, complex formation between p300 and BRG1 may be important for this acetylation of H3K122 (40).

There are no reports of any direct relationships of H3K18, H3K23, H3K36, H3K56, H4K5, H4K8, H4K12, H4K16, and H4K20 with cardiac hypertrophy and HF. However, acetylation of H3K9 and H3K27 has been reported in cardiac hypertrophy and HF. Other acetylation sites need to be investigated further based on the affected gene region and stage of the disease. Furthermore, the detailed role of these histone acetylation sites in the promoter and enhancer regions in cardiac hypertrophy and heart failure during transcription remains to be elucidated. Therefore, the relationship between histone acetylation and the expression of genes involved in cardiac hypertrophy and heart failure needs to be studied in detail.

## Histone acetyltransferase in heart disease

Selected lysines are acetylated by specific biological processes, and the overall histone acetylation level is dynamically regulated by two enzyme families, HATs and HDACs (41, 42). In histone acetylation, HATs require acetyl-CoA as cofactors to catalyze the transfer of the acetyl group to the *ε*-amino group of the lysine side chain (43). HATs can be divided into five families: p300/CBP, Basal TF, GNAT, MYST, and NCoA (44). Importantly, changes in the formation of complexes involving HATs can alter the sites of acetylated histones. For example, complex formation between p300 and BRG1 increases during cardiac hypertrophy and HF, and the level of H3K122 acetylation in the globular domain increases (40). Furthermore, yeast Gcn5 alone acetylates free histones *in vitro*, but it must be combined with the Ada2 and Ada3 subunits within ADA and SAGA complexes to preferentially acetylate H2B in the nucleosome (45). This increase in histone acetylation levels and changes in histone acetylation sites are involved in the formation of HAT complexes and activation of HATs. Therefore, understanding the transcriptional regulatory mechanisms related to HATs is important for the treatment of HF. Acetylation of histones by p300 is involved in cardiac hypertrophy and heart failure (46, 47). Hypertrophic stress increases HAT activity and histone acetylation, as p300 is regulated by several proteins, including ERK1/2, Akt, and Cdk9 (3). However, for HATs such as GCN5, CBP, and PCAF, the relationship with histone acetylation is unclear, although an association between cardiac hypertrophy and heart failure has been reported (48–50).Curcumin, a natural product, inhibits histone acetylation by blocking the HAT activity of p300, thereby improving cardiomyocyte hypertrophy and HF (32). Curcumin has also been reported to inhibit heart failure with preserved ejection fraction in Dahl rats (47). Recently, curcumin-based derivatives and analogs have been shown to have beneficial effects in HF (3, 4). Anacardic and eicosapentaenoic acids improve HF by inhibiting the HAT activity of p300. Similar to curcumin, anacardic acid also inhibits the HAT activity of p300 and improves the progression of cardiac hypertrophy and HF by inhibiting the acetylation of H3K9 (31, 51). In addition, direct inhibition of p300 HAT by eicosatetraenoic acid suppresses myocardial infarction-induced HF (28). Metformin can inhibit phenylephrine-induced cardiomyocyte hypertrophy by inhibiting p300 in cultured cardiomyocytes; therefore, it can potentially be used for the treatment of patients with diabetes and HF (52). In contrast, L003 and C646, specific HAT inhibitors of p300, inhibit angiotensin-induced cardiac hypertrophy and cardiac fibrosis (53). Compounds, such as resveratrol, also improve cardiac hypertrophy and HF by activating HDACs, such as sirtuin (54). In summary, preventing histone acetylation by inhibiting the HAT activity of p300 or activating HDACs can aid in the treatment of cardiac hypertrophy and HF. Moreover, drugs targeting various epigenetic processes can inhibit the HAT activity of p300 or activate HDACs for the treatment of HF.

## Histone deacetylase in heart disease

HDACs reverse histone acetylation by HATs and restore the original unacetylated state of histone lysine. There are four classes of HDACs, all of which are complexed with proteins that exhibit low substrate specificity (55). HDACs are classified into four major classes: HDAC class I (HDAC 1, 2, 3, and 8), HDAC class IIa (HDAC 4, 5, 7, and 9), HDAC class IIb (HDAC 6 and 10), HDAC class III (SIRT1-7), and HDAC class IV (HDAC11) (6, 56). HDACs cause chromatin enrichment by deacetylating histones, blocking access to DNA, and inhibiting transcription (57). The relationship between non-histone proteins and HDACs has been studied in cardiovascular disease. HDAC class I promotes cardiac hypertrophy and heart failure in many cases, whereas HDAC1 and HDAC2 inhibit cardioprotective and anti-hypertrophic genes (58, 59). Increased expression of HDAC8 in the heart increases p38 phosphorylation and expression, inducing cardiac hypertrophy and fibrosis (60). Trichostatin A and valproic acid have been shown to inhibit cardiac hypertrophy by inhibiting HDAC class I, making HDAC inhibition an attractive therapeutic target for heart failure (61). While HDAC class II plays a role in suppressing cardiac hypertrophy and heart failure, HDAC class IIa (HDAC 4, 5, and 9) suppresses cardiac hypertrophy by forming a complex with the transcription factor Mef2 (6). Phosphorylation of HDAC class IIa by phosphatases such as calcium/calmodulin-dependent protein kinase 2 prevents its binding to Mef2 and activates gene transcription (62). In addition, HDAC6 is involved in tubulin acetylation, myofibril stiffness and skeletal muscle wasting in cardiac disease (63–65). However, few reports on cardiac hypertrophy and heart failure associated with HDAC class IIb and IV are available, and he role of these HDACs in the heart needs to be clarified in the future. HDAC class III, also known as the SIRT protein family, plays a role in cardiac homeostasis; SIRT1 and SIRT3 promote the deacetylation of PGC-1*α* and reduce cardiac hypertrophy by reducing oxidative stress (66–68). SIRT2 exerts cardioprotective effect by promoting AMPK activation through deacetylation of LKB1 (69). SIRT6 inhibits cardiac hypertrophy by suppressing NFATc4 expression and activation (70). In contrast, a direct association between histone acetylation and HDACs in the heart has been reported. SIRT3 is involved in H3K27 deacetylation and regulates inflammation and fibrosis in the heart *via* modulation of the FOS/AP-1 pathway (71). SIRT6 binds to and represses the promoters of IGF signaling-related genes by deacetylating H3K9 through interaction with c-Jun. SIRT6 expression is downregulated in human heart failure, indicating that SIRT6 is involved in the pathogenesis of cardiac hypertrophy and heart failure (72). As mentioned above, few studies have demonstrated a direct relationship between HDACs and histone deacetylation in cardiac hypertrophy and heart failure. Therefore, the relationship between histone acetylation sites and HDACs should be studied in detail. It is also necessary to determine whether HDACs are negative or positive regulators of cardiac hypertrophy and heart failure.

## Conclusions and perspectives

**Figure 1:**
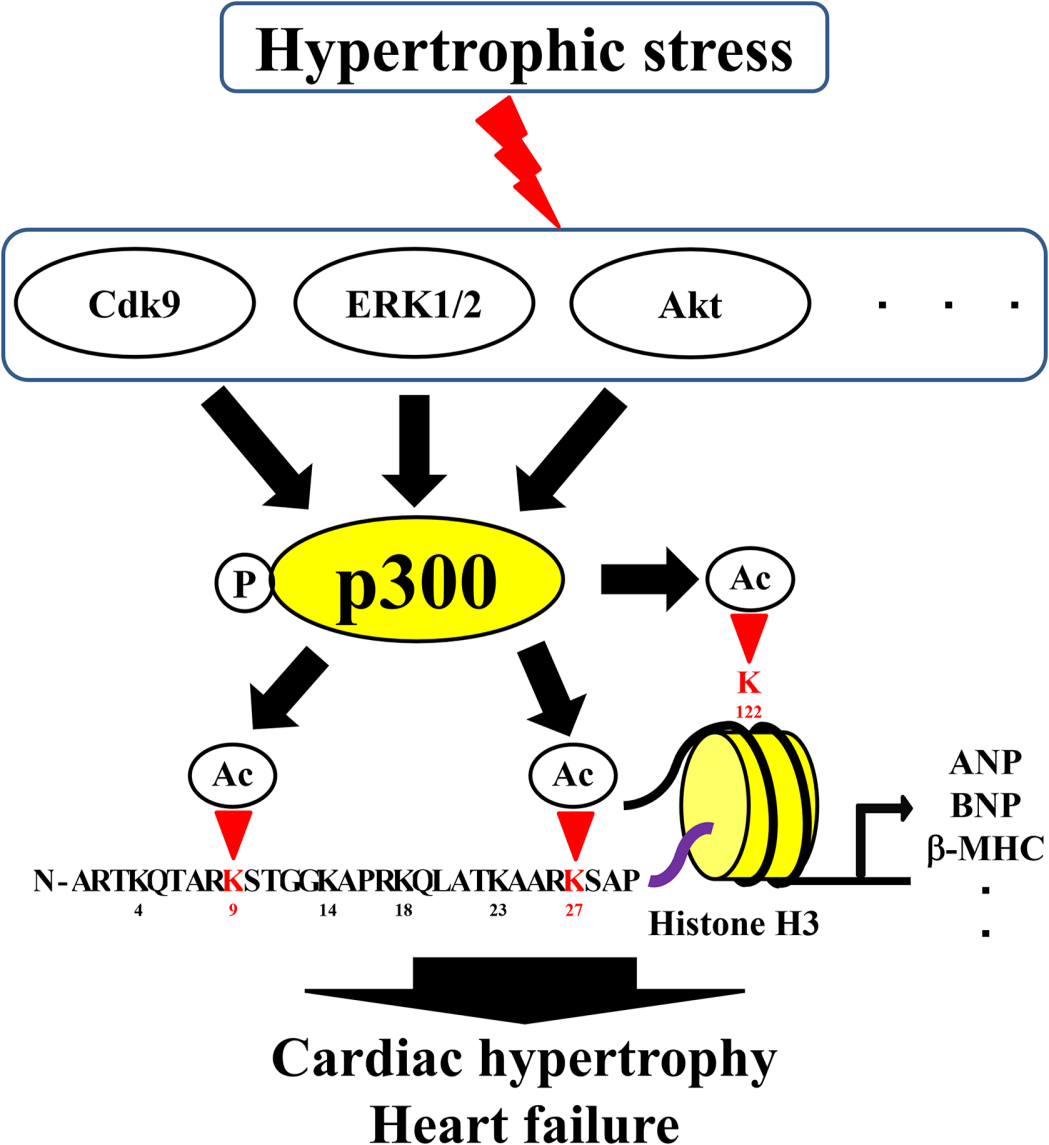
Roles of histone acetylation sites in cardiac hypertrophy and heart failure. Ac, acetylation; HATs, histone acetyltransferases; P, phosphorylation.

Histone acetylation and HAT are therapeutic targets, and their proper regulation is a promising therapeutic strategy for HF; however, some problems should be mentioned. Currently, only a limited number of histone acetylation sites have been implicated in HF (Figure 1). Therefore, future studies should investigate other histone acetylation sites throughout the genome. As the sites of histone acetylation are not similar in all gene regions, the overall map of histone acetylation sites in HF and the related HAT-centered transcriptional regulatory mechanisms need to be clarified. Since histone acetylation is regulated by the balance between HATs and HDACs, it is necessary to develop therapeutic agents targeting HDACs (9, 73). Although histone acetylation has been associated with gene expression during heart failure, there are also genes whose expression is altered independently of histone acetylation (9, 36, 40). Histone acetylation is associated with other epigenetic mechanisms such as histone methylation and DNA methylation (35). Therefore, the relationship between histone acetylation and other epigenetic mechanisms in cardiac hypertrophy and heart failure should be further investigated. In addition, post-translational modifications of histones, such as acetylation, in patients with heart failure have rarely been examined. Increased HAT activation of p300 has been reported in patients with heart failure (74); however, the association between histone acetylation and the development of heart failure in humans needs to be clarified.

In summary, epigenetic regulation by histone modifiers is critical in the pathogenesis of cardiac hypertrophy. Identifying the molecular mechanisms underlying the roles of histone modifiers in normal and disease states is essential for the development of novel “epigenetic” drugs to treat cardiac hypertrophy. Furthermore, as different histone acetylation sites have different effects on transcription, identifying time-specific acetylation sites in disease states is also important for the development of novel therapeutic agents for HF.
